# New strategies for neuro protection in glaucoma

**DOI:** 10.3389/fcell.2022.983195

**Published:** 2022-09-15

**Authors:** Yang Xuejiao, Yan Junwei

**Affiliations:** ^1^ Department of Ophthalmology, The Affiliated Hospital of Qingdao University, Qingdao, China; ^2^ Department of Vascular Surgery, The Affiliated Hospital of Qingdao University, Qingdao, China

**Keywords:** retinal ganglion cells, glaucoma, neuroprotection, gliocyte, gene therapy

## Abstract

Glaucoma is a progressive, irreversible loss of retinal ganglion cells (RGCs) and axons that results in characteristic optic atrophy and corresponding progressive visual field defect. The exact mechanisms underlying glaucomatous neuron loss are not clear. The main risk factor for glaucoma onset and development is high intraocular pressure (IOP), however traditional IOP-lowering therapies are often not sufficient to prevent degeneration of RGCs and the vision loss may progress, indicating the need for complementary neuroprotective therapy. This review summarizes the progress for neuro protection in glaucoma in recent 5 years, including modulation of neuroinflammation, gene and cell therapy, dietary supplementation, and sustained-release system.

## Introduction

Glaucoma is a progressive, irreversible loss of retinal ganglion cells (RGCs) and axons that results in a characteristic optic atrophy and a corresponding progressive visual field defect. The most common types of glaucoma are primary open-angle glaucoma and primary angle closure glaucoma (PACG) (Dietze et al., 2022). Acute PACG has typical anatomical characteristics, such as shallow anterior chamber, pupillary block, plateau iris, etc., it usually causes acute attack. However, patients with POAG and chronic PACG are often asymptomatic until the optic nerve damage is severe. The exact mechanisms underlying glaucomatous neuron loss are not clear.

Although some scholars believe that it is a neurodegenerative disease (Ramirez et al., 2017), it is not exactly the same as Parkinson’s disease, Alzheimer’s disease, and other neurodegenerative diseases that mainly occur in the middle-aged and the elderly. Glaucoma has a wide age, some young and middle-aged patients with open and chronic closure have very late visual field, obvious C/D cupping and optic nerve atrophy. Because irreversible blindness seriously affects patients’ quality of life and heavy social burden, it is critical to explore the possible pathogenesis of optic nerve injury and effective treatment targets.

The main risk factor for glaucoma onset and development is high intraocular pressure (IOP), and the current treatments available target the lowering of IOP (Dietze et al., 2022). However, degeneration of RGCs and the vision loss may progress despite significant IOP lowering in some patients, indicating that complementary neuroprotective therapy are needed. In recent years, a large number of studies on optic nerve protection have emerged, this review summarizes the progress for neuro protection in glaucoma in recent 5 years, including modulation of neuroinflammation, gene, and cell therapy, dietary supplementation, and sustained-release system.

## Neuroimmunity

Immune system dysregulation is increasingly being attributed to the development of a multitude of neurodegenerative diseases (Stothert and Kaur 2021). In recent years, a large amount of studies focus on the glia cells and immune system in the development of glaucomatous optic neuropathy (de Hoz et al., 2018). An excessive microglial response may be a significant degenerative factor for increased cell death (Grotegut et al., 2020), microglia activation and release of pro-inflammatory cytokines are the main contributors for retinal cell death in glaucoma (de Hoz et al., 2018). OPN was found to enhance the proliferation and activation of retinal microglia, and contribute to the eventual RGCs loss and vision function impairment in glaucoma (Yu et al., 2021). Blocking microglial A2A R prevents microglial cell response to elevated pressure and it is sufficient to protect retinal cells from elevated pressure-induced death (Aires et al., 2019). Another study found activation of Adenosine A (3) receptor could hinder the microglia reactivity (Ferreira-Silva et al., 2020), attenuated the impairment in retrograde axonal transport, and afford protection against glaucomatous degeneration. In addition, P2X7 receptor antagonist protects retinal ganglion cells by inhibiting microglial activation in a rat chronic ocular hypertension model (Dong et al., 2018).

Astrocytes perform critical non-cell autonomous roles following CNS injury that involve either neurotoxic or neuroprotective effects. Astrocyte-derived lipoxins A4 and B4 promote neuroprotection from acute and chronic injury neuroprotective signal (Livne-Bar et al., 2017). Statins promotes the survival of RGCs by reduce apoptosis and suppress chronic high IOP induced glial activation (Kim et al., 2021).

## Gene therapy

Gene therapy, which uses a viral vectors to deliver genetic material into cells, is a promising approach to directly target pathogenetic molecules (Keeler et al., 2017). The retina is a favorable target for gene therapy because of its easy access, established clear functional readouts, partial immune privilege and confined non-systemic localization (Ratican et al., 2018). The success of adeno-associated virus (AAV)-mediated gene replacement therapy for inherited retinal disease (Maguire et al., 2008; Busskamp et al., 2010; Smalley 2017) has made RGC-specific gene expression and AAV editing a promising gene therapy strategy for optic neuropathies. Table 1 lists the gene therapy studies on neuroprotection of glaucoma in recent 5 years, their findings indicate that gene therapy has a broad prospect in protecting both structure and function of RGC. Apart from this, reprogramming cells with defined factors is another promising strategy to produce functional cells for therapeutic purposes (Wang et al., 2021). OSK-induced reprogramming in mouse RGC was found to promote axon regeneration and reverse vision loss (Lu et al., 2020). Math5 and Brn3b transcription factors (TFs) combination can reprogram mature mouse Müller glia into RGC, resulting in proper projection of RGC in the visual pathway, and improved visual function (Xiao et al., 2021). Recently, another study, using a CRISPR-Cas9-based genome-wide screen of 1,893 TFs, found that manipulation of ATF3/CHOP and ATF4/C/EBPγ protected RGC in a glaucoma model (Tian et al., 2022).

**TABLE 1:** Gene therapy studies on neuroprotection of glaucoma in recent 5 years.

Target gene	Effect	Model	Function	References
Complement C3	overexpression of C3 inhibitor reduce the activation of complement C3d	intravitreal injection in mice glaucoma model	neuroprotection of retinal ganglion cells (RGC) axons and somata	Bosco et al. (2018)
Brain-derived neurotrophic factor (BDNF) and its receptor	increase the production of BDNF and TrkB	intravitreal injection in experimental glaucoma or humanized tauopathy model	improve long-term neuroprotective signaling, RGC survival, and functional recovery	Osborne et al., 2018, Wojcik-Gryciuk et al., 2020, Khatib et al., 2021
Vascular endothelial growth factor (VEGF)	transduction of VEGF variants by VEGFR2 and PI3K/AKT signaling	AAV2-mediated transduction into primary mouse RGC	promote synaptogenesis, increase the length of neurites, axons	Shen et al. (2018)
γ-synuclein (mSncg) promoter	combine AAV-mSncg promoter with CRISPR/Cas9 gene editing knock down pro-degenerative genes	AAV2-mSncg in hPSC-derived RGCs and mice ON crush model	preserve the acutely injured RGC somata and axons	Wang et al. (2020)
CaMKII	increase the expression level of CaMKII	intravitreal injection AAV for the treatment of CaMKIIα T286D in a mouse model of glaucoma	protection of RGC and their axons	Guo et al. (2021)
BCLX_L_	gene therapy with mCherry-BCLX_L_ and force its overexpression	intravitreal injection in mice glaucoma model	robustly attenuate both RGC soma pathology and axonal degeneration in the optic nerve	Donahue et al. (2021)
NMNAT	overexpression of NMNAT2 mutant driven by mSncg promoter restore the decreased NAD + levels	intravitreal injection in mice glaucoma model	significant neuroprotection of both RGC soma and axon and preservation of visual function	Fang et al. (2022)
Myc-associated protein X (MAX)	gene therapy by overexpression of MAX	intravitreal injection in rat glaucoma model	prevent RGC death and protect optic nerve axons	Lani-Louzada et al. (2022)
X-linked inhibitor of apoptosis (XIAP)	blocking the activation of apoptosis	intravitreal injection in mice glaucoma model	provide both functional and structural protection of RGC	Visuvanathan et al. (2022)

## Cell therapy

Cell therapy provides a new therapeutic strategy for glaucoma. Stem cell therapy mainly involves the transplantation of cells to replace the dead and lost RGC. However, it is associated with a number of major challenges besides ethical issue. Regeneration of RGCs requires full synaptic integration of host inner retinal stem cells and the development of long-distance axons, which project to the brain and accurately form effective synaptic connections with corresponding targets to complete signal transmission. Up to now, the replacement of RGCs has not made a breakthrough (Zhang J. et al., 2021). Several recently studied cell types for transplantation including mouse induced pluripotent stem cell (miPSC) or mouse embryonic stem cell (mESC)-derived RGC (Oswald et al., 2021) and spermatogonial stem cell-derived RGC (Suen et al., 2019). Another study found mesenchymal stem cells (MSC) secreted exosomes can promote survival of RGC and regeneration of their axons (Mead and Tomarev 2017). In addition, further study found that TNF-α stimulated gingival MSC derived exosomes play neuroprotection and anti-inflammation roles by delivering miR-21-5p-enriched exosomes through MEG3/miR-21-5p/PDCD4 axis (Yu et al., 2022).

## Dietotherapy

In animal models of glaucoma, various diet-related treatments were found as non-IOP-related neuroprotective mechanisms. High VitK1 intake (Deng et al., 2020) , Coenzyme Q10 + Vitamin E (Zhang et al., 2017; Ekicier Acar et al., 2020), Nicotinamide riboside of the vitamin B3 family (Zhang X. et al., 2021), probiotic bacteria (Fafure et al., 2021) and other dietary supplementation (Cammalleri et al., 2020) were proved to attenuate the loss of RGCs by regulating glia-mediated neuroinflammatory or BDNF activity, etc.

## New drug loading system

Several preclinical studies demonstrate that neurotrophins (NTs) prevent RGCs loss (Gupta et al., 2022). NTs can be conjugated to nanoparticles, which act as smart drug carriers. This enables the self-localization of drugs in the retina and the prevention of rapid degradation of drugs (Giannaccini et al., 2018).

Sunitinib is a protein kinase inhibitor with activity against the neuroprotective targets dual leucine zipper kinase (DLK) and leucine zipper kinase (LZK). It was found to enhance survival of RGCs for neuroprotection. Recently, a hypotonic, thermosensitive gel-forming eye drop (Kim et al., 2022) and a sunitinib-pamoate complex (SPC) microcrystals for subconjunctival injection (Hsueh et al., 2021) were devised to continuously release for 1 and 20 weeks.

## Others

Cannabinoids (CBs) was found to target several factors that related with the progression of glaucoma, it promotes neuroprotection, abrogates changes in ECM protein, and normalizes the IOP levels in the eye (Maguire et al., 2022; Somvanshi et al., 2022).

**FIGURE 1:**
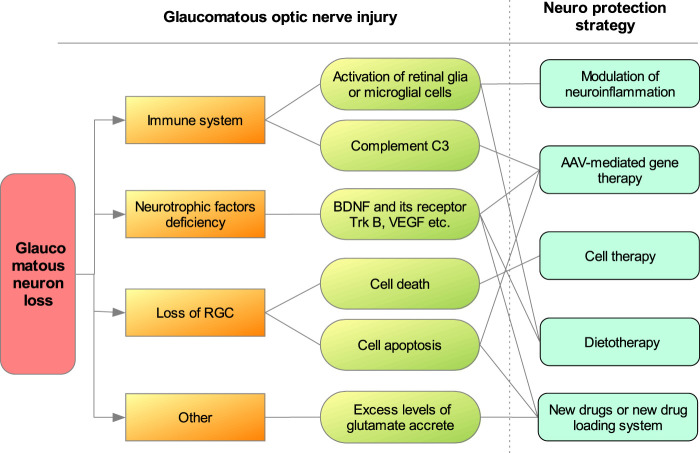
New neuroprotective strategy of glaucoma in recent 5 years.

In summary, various neuroprotective therapy (Figure 1) can help us to better understand the pathological basis of visual function impairment and progression in glaucoma. At present, many scholars have committed to clinical translation to save RGCs and visual function of glaucoma patients from the molecular and cellular levels. These new strategies will bring hope for the prevention and treatment of glaucoma.
